# Perceived barriers and facilitating factors prior to the implementation of a lifestyle focused approach in the treatment of inpatients with mental illness

**DOI:** 10.1192/j.eurpsy.2022.1577

**Published:** 2022-09-01

**Authors:** M. Van Schothorst, N. Den Bleijker, N. De Vries, P. Van Harten, J. Deenik

**Affiliations:** 1 GGz Centraal, Scientific Research Department, Amersfoort, Netherlands; 2 Maastricht University, School For Mental Health And Neuroscience, Maastricht, Netherlands

**Keywords:** Lifestyle, Implementation, mental illness, barriers and facilitators

## Abstract

**Introduction:**

Despite the increasing evidence for the efficacy of lifestyle interventions for people with mental illness (MI), there has been little change in routine clinical care. There are several factors that can complicate or facilitate the implementation of a lifestyle intervention. Gaining insight into such factors can contribute to effective integration into clinical practice.

**Objectives:**

To assess the perceived barriers and facilitating factors of healthcare professionals (HCPs) and inpatients of psychiatric wards for the use of a lifestyle focused approach, prior to its implementation.

**Methods:**

Baseline data from an open cohort cluster randomized stepped wedge study. Barriers and facilitators with regards to the intervention, HCPs and inpatients, and the organization were assessed with the measurement instrument for determinants of innovations, online, or through a semi-structured interview.

**Results:**

Initial results show that inpatients (N=167) experience both barriers and facilitating factors with regards to themselves and the innovation. They perceive the innovation as complex and see few personal benefits, but indicate that they consider it part of their treatment. Healthcare workers (N=77) perceive facilitating factors related to themselves and the organization, such as expected support, but were not sufficiently aware of the content of the innovation. More detailed exploration of relationships with demographic and disease-related factors are currently being conducted.

**Conclusions:**

These findings provide insight into the perceived barriers and facilitators of inpatients and HCPs regarding a lifestyle focused approach, prior to its implementation. More insight into relationships with demographic and disease-related factors can benefit application into routine clinical care.

**Disclosure:**

No significant relationships.

